# Supplementary material for: Common Peroneal Nerve Paralysis Following Rapid Weight Loss—A Case Report and Literature Review
Source: Nutrients. 2025 May 24;17(11):1782. doi: 10.3390/nu17111782 (PMC12157737; doi:10.3390/nu17111782)
Supplement: Supplementary file 1 [file nutrients-17-01782-s001.zip › Figure S1.pdf]

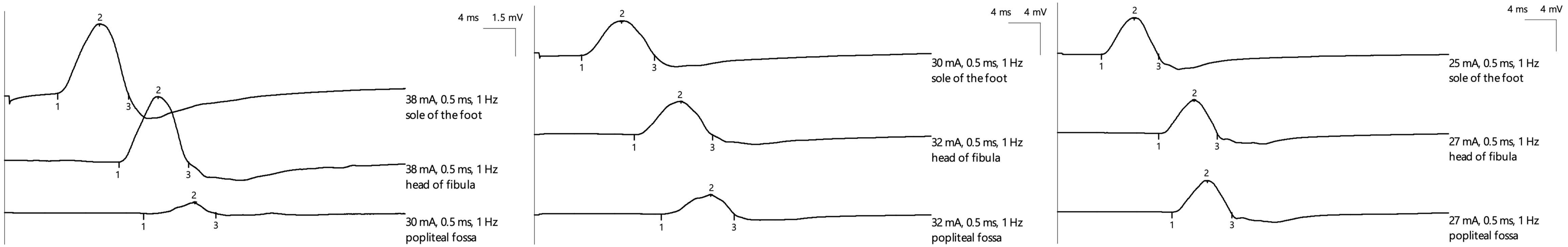

A.

Pre-treatment **2-month follow-up** **4-month follow-up**

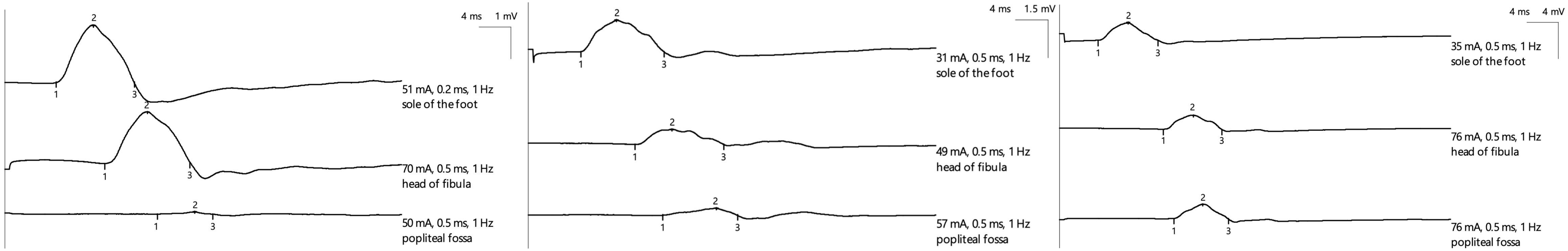

B.

Pre-treatment **2-month follow-up** **4-month follow-up**

Figure S1. Peroneal nerve conduction study recording from extensor digitorum brevis muscle (L4-L5-S1). Right (A) and left (B) lower extremities. Follow-up evaluation reveals decreased latency and increased motor response amplitude consistent with nerve recovery.
