# Supplementary material for: Common Peroneal Nerve Paralysis Following Rapid Weight Loss—A Case Report and Literature Review
Source: Nutrients. 2025 May 24;17(11):1782. doi: 10.3390/nu17111782 (PMC12157737; doi:10.3390/nu17111782)
Supplement: Supplementary file 1 [file nutrients-17-01782-s001.zip › tables_supplementary data.pdf]

Table S1 – General data regarding articles included in the systematic review

Study	No of cases	Cause of weight loss	General data of patients	Clinical presentation	Blood tests	Diagnostic investigations	Treatment	Outcome
Elias et al, 2006 [17]	n = 1	BS	53y, F, -39.5 kg in 6 mo and 51.8kg in 1 year (36.83% from the initial BW)	Right foot drop 6 mo and left foot drop 1y after bariatric surgery	Albumin, ferritin, folate, vit. B12 - N<br>DM	Lumbar MRI:<br>Knee and thigh MRI<br>EMG/NCS	Surgical	Partial recovery after 2 mo
Pennington, 2024 [19]	n = 1	GLP-1 agonist treatment	49y, F, -31.8 kg in 6 mo	Sudden onset of right foot drop	No DM	Right knee X-rays<br>EMG/NCS<br>Knees MRI	Conservative	Complete recovery in 1 mo
Tucker and Ritchie, 2024 [20]	n = 2	Tirzepatide treatment	77y, M, -27.6 kg in 7 mo (24% from the initial BM)<br>56y, F, -15.8 kg in 1y (28% from the initial BW)	Bilateral foot drop	CBC, CMP, TSH, SPEP, vit. B1, B6, B12, heavy metals, cooper, TP Ab – N<br>Elevated copper (n=1)<br>Low Vit B6 (n=1)<br>Prediabetes (n=1)<br>DM (n=1)	Lumbar MRI (n=2)<br>EMG/NCS (n=2)<br>Knee US (n=1)	Surgical	Clinical improvement (n=1)<br>Not mentioned (n=1)
Baharin et al, 2022 [21]	n = 1	Diet	19y, F, -20 kg in 4 mo (19.6% from the initial BW)	Bilateral foot drop	Fasting blood sugar, thyroid function test, thiamine, folate, vitamin B12, ANA, complement levels and anti-dsDNA- N	EMG/NCS	Conservative	Complete recovery after 3 mo
Özişler et al, 2017 [22]	n = 1	BS	30 y, F, -40 kg in 16 weeks (44% from the initial BW)	Left foot 18 weeks and right foot 24 weeks after bariatric surgery	Glucose and lipid metabolism parameters - N; no nutritional deficiencies (not mentioning the exact tests)	EMG/NCS	Conservative	Not mentioned (Contact with patient lost)
Prado, 2010 [23]	n = 2	BS	49 y, M, -45 kg in 11 mo (54% from the initial BW)<br>22 y, F, -38 kg in 6 mo (41.04% from the initial BW).	Bilateral foot drop<br>Right foot drop	Blood tests, vitamins, minerals – N (not mentioning the exact tests).	MRI (n=1) (not mentioning the location)	No treatment (n=1)<br>Not mentioned (n=1)	Spontaneously recovery in 45 days (n=1)<br>Complete recovery in 3 weeks (n=1)
Oh et al, 2021 [24]	n = 1	Biliary surgery	58y, M, -10 kg in 24 days (14.7% from the initial BM)	Bilateral foot drop.	Thyroid function test, Vit. B1, B6, B12, folate - N<br>Without DM.	EMG/NCS<br>Lumbar MRI<br>Lower limbs US<br>Knees MRI	Conservative	Clinical improvement in 4 weeks
Cruz-Martinez et al, 2000 [25]	n = 30	Intentional weight loss.	27.6 ± 9.79 y<br>18 F, 12 M<br>-10.9 ± 5.27 kg in 2 weeks to 12 mo	Left foot drop (n=16)<br>Right foot drop (n=14)<br>Sudden onset (n=30)	Other polyneuropathies or systemic disease excluded	EMG/NCS (n=30)	Conservative (n=29)<br>Surgical (n=1)	Clinical improvement in 3 weeks – 3 mo (n=29)<br>Clinical improvement in 2 weeks
Şen et al, 2019 [18]	n = 7	BS	-50.6 kg in 6 months (60.2 ± 19.6% from the initial BW).	Bilateral foot drop (n=1)<br>Unilateral foot drop (n=6), not mentioning the right or left side	No nutritional and vitamin deficiency (not mentioning the exact tests).	EMG/NCS (n=7)	Surgical (n=3)<br>Conservative (n=4)	Complete recovery in 2 mo (n=1). Not mentioned for the others (n=3).<br>Complete recovery. How long it is not specified.
Lale et al, 2020 [26]	n = 31	BS	Mean age 35.5±10.4 y<br>64.3% F<br>-72.8±21.5% from the initial BW in 6 mo and 92.3±25.9 from the initial BW in 1 y	Unilateral symptoms (n=31), not mentioning the right or left side	No vitamin or mineral deficiency (not mentioning the exact tests).	EMG/NCS (n=21)<br>US (n is not mentioned)<br>Knee MRI (n is not mentioned)<br>Lumbar MRI (n is not mentioned)	Conservative (n=4)<br>Surgical (n=9)<br>Not mentioned (n=20)	2 patients with no improvement after 2 weeks of conservative treatment underwent surgery<br>Clinical improvement 2 mo after the surgery
Meylaerts et al, 2011 [27]	n = 5	Intentional weight loss	19-79 y, 60% F, -8-17% of initial BW	Not mentioning the exact number of uni or bilateral foot drop.	Not mentioned	EMG/NCS (most cases)<br>Knee MRI(most cases)<br>Knee US (n=5)	Surgical	Partial recovery (n=4). How long it is not specified
Weyns et al, 2007 [36]	n = 78	Diet, BS, severe illness, psychiatric disorders	27-48 y, 77.77% F<br>-45 kg in 8.6 mo (38.3% from the initial BW)	Foot drop (without mentioning uni or bilateral).	Not mentioned	EMG/NCS (n=78)	Surgical (n=78)	Clinical improvement (n=78). How long it is not specified.
Shields et al, 2021 [11]	n = 5	Diet<br>Exercise,<br>Hospitalization	65y, F, -41 kg in 1 y<br>31y, M, -45 kg in 26 days<br>43y, F, -32 kg in 1 y<br>51y, F,-32 kg in 1 y<br>71, F, -57 kg in several mo	Left foot drop (n=1)<br>Right foot drop (n=3)<br>Bilateral foot drop (n=2)	DM (n=2)	EMG/NCS (n=5)<br>Lumbar MRI (n=3)<br>Knee MRI (n=2)<br>Knee US (n=2)	Surgical (n=2)<br>Methylprednisolone (n=1)<br>Conservative (n=2)	Clinical improvement after 2 weeks and 1 mo<br>Improvement<br>Improvement (n=1)<br>Not mentioned (n=1)
Aguirre et al, 2022 [29]	n = 1	Diet and long-distance walking	56y, F, -49 kg in 1y (41.28% of her initial BW)	Bilateral foot drop	CBC, A1c%, vit.D, B12, iron, Ca, AST, ALT, TIBC, Mg, genetic tests for neuropathies and neuromuscular disorders - N	EMG/NCS<br>Right knee MRI	Conservative	Complete recovery in 1 y

Broekx and Weyns, 2018 [30]	n = 200	24.5% unknown, 15.5% BS 5.5% diet	Mean age 55 y<br>58% M<br>-19.43 kg	Right foot drop (52%).<br>Not mentioning the exact number of uni or bilateral foot drop.	Not mentioned	EMG/NCS (n=200)<br>Knee US (n=200)<br>Knee MRI (n=61)<br>Spinal MRI (n=17)	Surgical (n=200)	Clinical improvement in 83 days to 3 mo<br>3.5% with no motor improvement<br>1.89% with no sensory improvement
Papagianni et al, 2008 [31]	n = 9	Starvation from appetite loss	Mean age = 46.8y<br>Sex = 90% male<br>-10-20% of their initial BW	Right foot drop (n=5)<br>Left foot drop (n=3)<br>Bilateral foot drop (n=1)	well-controlled DM (n=2)	EMG/NCS (n=9)	Not mentioned	Not mentioned
Margulis et al., 2018 [32]	n = 1	Diet	60y, M, -40 kg in 1y	Back pain<br>Bilateral foot drop	Vit. B1, B12, E - N<br>Other polyneuropathies excluded	EMG/NCS<br>Lumbar CT<br>Knees X-rays<br>Knees MRI	Dexamethasone<br>Conservative<br>Surgical after 6 mo	-Decreased back pain, no improvement in weakness and hypesthesia<br>-Full recovery in 12 mo
Ramos-Levi et al, 2013 [33]	n = 2	BS	30y, F, -44.3 kg in 6 mo (36% from the initial BW).<br>45y, M, -46.2 kg in 6 mo (35.4% from the initial BW).	Right foot drop (n=2)	Total proteins, albumin, prealbumin, iron, folic acid, vit B12, Zinc, Selenium, Copper, retinol, vit E/cholesterol – N; low 25-OH vit D (n=1)	EMG/NCS (n=2)	Conservative	Complete recovery after 6 mo and 1 y
Saritosun et al, 2025 [34]	n=1	BS	50y, F, -30kg in 7 mo,	Right foot drop	DM<br>Glucose, creatinine, thyroid function test, vit B12, folate	Lumbar MRI<br>Knee US<br>EMG	Conservative	Almost complete recovery after 4 mo
Lee et al, 2024 [35]	n=1	BS	48y, F, -23 kg in 3 mo and – 46kg in 11 mo	Right foot drop	No DM<br>CBC, vit B12, folate, calcium, albumin, ferritin, C-reactive protein, liver enzymes, renal function, thyroid function test<br>Borderline normocytic anemia	Knee X-rays<br>Knee US<br>EMG<br>Knee MRI<br>Mild denervation changes in tibialis ant	Conservative<br>Vit B12	Complete recoveruy in 1.5y

Abbreviations: A1c% - glycated hemoglobin, ANA - Antinuclear Antibody, anti-dsDNA – anti-double stranded DNA, BS – bariatric surgery, BW – body weight, CBC - complete blood count, CMP - comprehensive metabolic panel, DM - diabetes mellitus, EMG/NCS - Electromyography/Nerve Conduction Studies, mo - month/months, MRI - magnetic resonance imaging, SPEP - serum protein electrophoresis, N – Normal, TP Ab - Treponema pallidum antibodies, TSH - thyroid-stimulating hormone, US – ultrasonography, vit-vitamin, y - year/years.

Table S2 - Demographic characteristics and bariatric procedures in patients who developed peroneal nerve palsy following bariatric surgery

Study	Country	No of cases	Patient characteristics	Bariatric surgery procedure
Elias et al, 2006 [17]	United States of America	n = 1	53y, F	Roux-en-Y gastric bypass
Özişler et al, 2017 [22]	Turkey	n = 1	30y, F	Gastric banding
Prado, 2010 [23]	Brazil	n = 2	49 y, M<br>22 y, F	Gastroplasty (n=1)<br>Gastroplasty with cholecistectomy (n=1)
Şen et al, 2019 [18]	Turkey	n = 7	3 M<br>3 F<br>Mean age 43 (n=6)	Laparoscopic sleeve gastrectomy
Lale et al, 2020 [26]	Turkey	n = 31	Mean age 35.5±10.4 y<br>64.3% F	Laparoscopic sleeve gastrectomy (n=8)<br>One anastomosis gastric bypass (n=1)<br>For the rest of the patients (n=22) the exact procedure is not mentioned
Weyns et al, 2007 [36]	Belgium	n = 9	27-48 y<br>77.77% F	Gastric banding n=5<br>Gastric bypass n=4
Broekx and Weyns, 2018 [30]	Belgium	n = 31	Not mentioned	Not mentioned
Ramos-Levi et al, 2013 [33]	Spain	n = 2	30y, F<br>45y, M	Vertical gastric gastroplasty (n=1)<br>Laparoscopic biliopancreatic diversion with duodenal switch (n=1)
Saritosun et al, 2025 [34]	Turkey	n=1	50y, F	Sleeve gastrectomy
Lee et al, 2024 [35]	New Zealand	n=1	48y, F	Sleeve gastrectomy
